# Assessing the Mediating Role of Safety Communication Between Safety Culture and Employees Safety Performance

**DOI:** 10.3389/fpubh.2022.840281

**Published:** 2022-03-10

**Authors:** Gehad Mohammed Ahmed Naji, Ahmad Shahrul Nizam Isha, Abdulsamad Alazzani, Muhammad Shoaib Saleem, Mohammed Alzoraiki

**Affiliations:** ^1^Department of Management and Humanities, University of Technology Petronas, Tronoh, Malaysia; ^2^Department of HRM, College of Administrative and Financial Science, Qatar University, Doha, Qatar; ^3^Gulf University, Sanad, Bahrain

**Keywords:** safety culture, safety communication, safety performance, petrochemical sector, Malaysia

## Abstract

The main purpose of this research was to investigate the mediating role of safety communication (SCO) in the relationship between safety culture (SC) and safety performance (SP) amongst employees in the petrochemical industry. Safety communication methods not only enhance working conditions but also have a positive impact on employee's behaviors and attitudes toward safety leading toward reduced incidents in the workplace environment. A stratified sampling method was followed to collect data in the petrochemical industry in Malaysia. Structural equation modeling (SEM) was utilized to analyze the hypothesized model, using data from 320 participants. The findings reveal that safety communication partially mediates the association between safety culture and safety performance. Further, safety culture was found to have a significant and positive effect on safety performance. This -study makes a significant theoretical contribution by providing empirical evidence on the direct and indirect relationship between safety culture and safety performance in the petrochemical industry.

## Introduction

Despite a wide variety of approaches and ideas throughout the years to enhance safety culture and occupational health and safety in the workplace, the petrochemical oil and gas industry has faced difficulties to diminish a high rate of fatalities and injuries amongst working staff (1, 2). In the United States alone there were 138 deaths, due to of the nature of work in the petrochemical industry (3), and this regrettable trend extended throughout the sector's operations around the world. By comparison, the industry's on-the-job fatality rate in the United States was ~7.6 times higher than the national average (4).

Many previous studies have examined the link between safety culture and safety outcomes (5–9). However, there is a lack of evidence in the literature on the essential role of safety communication in the relationship between safety culture and safety performance. Effective safety communication plays a vital role in reducing incidents of employees at the workplace. Safety communication is not only about the exchange of information on safety in the workplace; it is also about influencing staff behavior and attitudes toward safety (10). According to Noort, Reader, Shorrock, and Kirwan (11) effective safety communication positively affects safety performance. On the other hand, Mullen (12, 13), identified that poor communication often occurs among workers, particularly among employees and senior management, and may be due to a neglect of constructive communication and feedback regarding workplace safety. One of the reasons for poor safety communication is the lack of a positive safety culture (14). Therefore, the current study aims to fill this gap and contribute to safety literature, by investigating the role of safety communication as a mediating factor in the relationship between safety culture and employee safety performance.

This study focuses on the petrochemical and oil and gas industry since this entails high risks related to health and safety. The oil and gas sector is under public scrutiny like never before on a host of health, safety, and environmental problems (15). These concerns are already impacting how companies operate and interact with the public. A pilot program with nine companies called the SafeOCS Industry Safety Database managed by the Bureau of Transportation Statistics in the U.S, has selected three potential next steps for the use of this industry wide safety database. One of the main steps is: “Develop effective communication processes, including dashboards, to share lessons learned, review aggregated results, assess causal factors, network, and discuss potential actions to prevent recurrence and thereby improve safety” (16). According to Curlee et al. (17), all the petrochemical and oil and gas production and exploration activities, typically experience the highest fatality rates of all the major industries. In the same line, Cloughley and Thomas (18), stated thatpetrochemical activities tend to be harsh and hazardous as they take place within ahigh pressure and high temperature (HPHT) environment.

Based on the aforementioned argument, we hypothesize that safety communication will mediate the relationship between safety culture and employee safety performance. Safety culture, by reducing accidents, allows employers to invest their resources in obtaining and enhancing safety performance (19–21). The rest of the paper is structured as follows: Section 2 provides a review of the literature and develops the study hypotheses; Section 3 presents the methods and materials; Section 4 presents the data analysis and results, Sections 5 and 6 present the discussion and conclusion.

All in all, this study aims to provide new theoretical and practical insights in the relationship between safety culture and safety performance by exploring the key role of safety communication as a mediating variable. It also does so in a newly industrialized country context.

## Literature Review

Personnel in the field have to deal with the instability of the drilling and production process. Activities such as overbalanced wells, gas leakages, and generally, the reactive nature of hydrocarbon resources tend to create life-threatening situations if not monitored well (22). That is why much of the literature has focused on the health safety environment (HSE) in the petrochemical, oil and gas sector.

Employers paid around “1 billion USD per week” for direct staff compensation costs according to National Safety Council statistics in 2010. The economic influence of workplace injuries in the U.S. were nearly 142 billion USD every year and the production loss because of accidents and injuries was equal to 80 million days lost every year (23). While deaths and injuries are irreversible effects, there are several financial and environmental effects involved as well. Indeed, if everybody behaved and performed safely, the production would certainly increase, or at least there would be no drop in production. A survey conducted by Liberty Mutual and cited by (24) found out amongst executives that:

❖ 9% indicated that a company's financial performance is greatly influenced by a safe workplace.
❖ 61% assumed that for each $1 for safety invested, a $3 or more return was observed.
❖ 13% stated that in each $1 invested in safety, $10 would be reimbursed.
❖ And 40% expected indirect costs between $3 and $5, for every $1 in direct costs of injuries or incidents.

From the devastating impact of the Bhopal tragedy in 1984 to the Macondo accident in the Gulf of Mexico in 2010, accidents have been proven to affect individuals, the environment, and the company's brand, as well as result in additional legal concerns such as criminal charges (25). The adverse effects of industrial incidents on societal loss have been evaluated in numerous studies (26).

**Figure 1:**
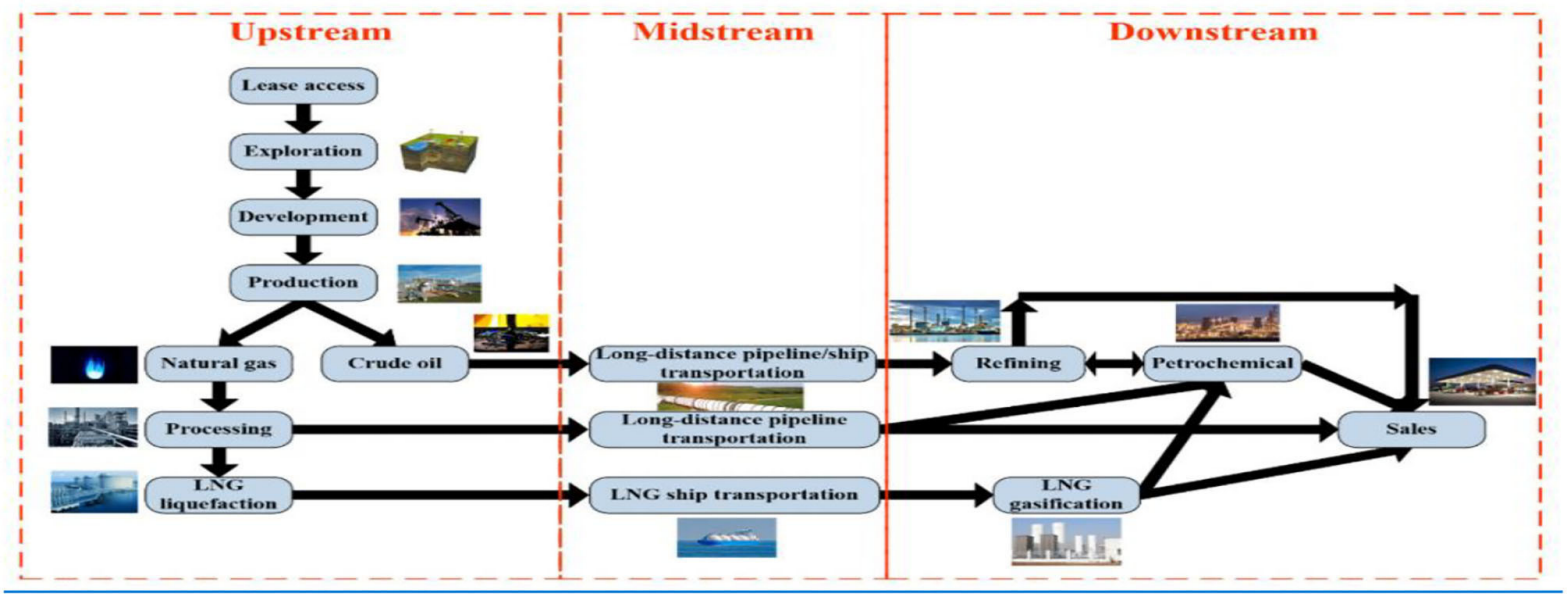
Upstream, midstream, and downstream operations in the oil and gas sector. Source (82).

There are three sectors in the petrochemical industry: upstream, midstream, and downstream. Employees in all three industries operate in high-risk conditions (Figure 1). The upstream sector is responsible for the production of fundamental raw materials, the midstream sector is for intermediates, and the downstream sector is in charge of the process and production of diverse byproducts (20).

### Upstream Operations

The upstream operation is the initial step in the oil and gas production process, and it includes searching for crude oil and natural gas deposits, as well as recovering and producing them (27). Within the oil and gas industry, this stage is known as the E&P (exploration and production) stage (28). Searching for oil reserves, either underground or underwater, drilling exploratory wells, and, if deposits are discovered, operating these wells to bring found deposits to the surface are all common areas in the upstream process.

### Midstream Operations

The natural gas and oil industry's midstream operation makes a significant contribution to everything needed to transport and store crude oil and natural gas before they are refined and processed into fuels and critical components for a long list of products used every day. Pipelines and all the infrastructure required to transport these resources over far distances, such as pumping stations, tank trucks, rail tank cars, and transcontinental tankers, are all part of the midstream industry (29, 30).

### Downstream Operations

The final and most diversified stage of the oil and gas industry is downstream operations. This could include everything from crude oil refining to natural gas processing and purification, as well as sales, marketing, product distribution, and retail. The production of petrochemicals and plastics is also included. During the downstream stage, many products derived from crude oil are produced, including diesel oil, liquefied petroleum gas (LPG), petrol, fertilizers, antifreeze, pharmaceuticals, and even cosmetics (31). Downstream is without a doubt the part of the oil and gas business that has the most direct relationship to daily consumers, which means that jobs in this sector are in high demand and diverse (32, 33).

### Safety Culture

Many companies throughout the world are becoming more interested in the concept of “safety culture” as a way to reduce the risk of large-scale disasters and accidents that occur during ordinary work. Its expanding relevance is evidenced by publicly declared goals in the offshore, shipping, and nuclear industries to achieve consistent global safety cultures (34–37). Companies are motivated to find solutions to prevent workplace injuries because of the costs connected with them and the time necessary for accident investigations (38, 39).

Improved culture and behaviors of operating personnel play a vital role in safe operations (40, 41). Visible leadership motivates personnel and enhances the performance of the company. It also enhances the commitment of the operating personnel. It has been observed that people are poor in assessing risks whilst performing operations as people tend to become acclimated to risk, i.e., “Used to Risk.” They unconsciously adjust their definition of acceptable risk. E.g., NASA Officials got used to living with small failures, the result-the catastrophic failure of the challenger. Overconfidence is also one of the main reasons for accidents–exposure to fairly fixed and no risk over a long period results in the underestimation of risk (42, 43). People have trouble imagining how small failings can lead to catastrophic disasters (Dominos Effect). Ignorance supplies fuel for accidents to happen. No matter how well defined a system is, someone will find a way to defeat the pro-active measures. A large number of cases are available in the industry (44). Many previous studies (45, 46), mentioned that there is a significant relationship between safety culture and safety communication.

As a result, the organizational culture, specifically the safety culture, is predicted to play a critical role in the mitigation of safety communication, hence hypothesis 1 was developed to investigate the link between safety culture and safety communication.

**Hypothesis 1:** Safety culture has a positive impact on safety communication.

### Factors of Safety Cultures

#### Work Environment

Workplace environment (WE) refers to the procedures and policies that must be implemented to ensure the health and safety of employees. The first policy entails accident detection and control based on regulatory regulations, as well as staff safety training and education (47). The immediate context in which an individual conducts his task is referred to as the workplace environment. The setting in which employees work has a significant effect on the quality of their work and their performance. Improper surroundings present dangers, making the workplace environment dangerous and slowing the employee's production rate (48, 49). Furthermore, health and safety at work have professionals and legal obligations to provide employees with a workplace free of hazards that could result in significant physical injury or death. In addition, they must provide a safe and healthy working environment for their employees.

#### Organization Communication

Organizational communication (OC): is known as a productive relationship with others, understanding and listening to each other. Conveying ideas and communications obviously and consistently; expressing things comprehensibly and simply, in ways that others can understand, and showing sincere knowledge, interest, and concern; bringing these features together to make variation happen. Communication effectively sustains the development of optimistic relationships with all investors and can impact attitudes and behaviors regarding safety and health issues (50).

#### Leadership

Leadership (LS) is related to the process of social influence, which maximizes the effects of others, toward the achievement of a goal. Leaders are individuals who have great influence attitudes and behaviors of others. Usually, they do this through their normal character and by their influence. Leaders can be assigned at any position in an organization from the board and senior executives, through middle-level managers such as site managers, to front-line supervisors (19, 51–53). Safety and health improvement in an organization depends on positive leadership and competent management.

### Safety Performance

Kucherov et al. (54), mentioned that safety performance occurs when front-line individuals improve their behaviors, however, behaviors on the front line will only change if there is a great leader to monitor their behaviors, make a different feeling of belonging in the teamwork. With this regard, oil and gas companies are increasingly developing and implementing safety leadership training programs. It aims to help leaders to create a personal and organizational safety imperative to be apart from their daily personnel behavior, provide Leaders to deliver the cultural and behavioral changes on the front line, and make safety leadership self-sustaining with the organization (55, 56). People, processes, tasks, and systems can interact purposefully and cooperatively to achieve health, safety, and environmental (HSE) goals through communication. The way we communicate with safety will have an impact on whether or not people accept and engage in the process, and the language we use will frequently indicate whether or not the method is accepted (9). Graphs representing lost time, medical procedures, employee's compensation rates, intensity and accident rates, and good performance measures can be used to communicate an organization's performance. These are used to enable continuous improvement and increase line employee's responsibilities for meeting an organization's HSE goals (57).

Safety incidents data have been collected and categorized by Oil and Gas Producers (OGP) and the international association of oil and gas producers since 1985. (IOGP) obtained the largest database of safety performance in the exploration and production industry. Around fifty participant organizations of the oil and gas industry contribute to annual benchmarking processes that focus mainly on incidents, accidents, and injuries in the oil and gas companies (58, 59).

According to Coday et al. (60), the process of extracting hydrocarbons in remote geographical locations and harsh environmental conditions is drastically increasing worldwide. Combined associations and established partnerships between giant oil producers on risky international projects are common. Sharing contracts with several investors must be managed properly. There is a high rate of non-productive time that requires swift and decisive action, and the overall equipment efficiency needs to grow (61, 62).

Human and environmental safety and health protection remain the number one priority for the oil and gas industry. These companies are used to dealing with stringent EHS regulations across the entire span of their activities, from exploration and production, to pipeline management and marketing (63, 64). These regulations are not only stringent but also constantly revised to take into consideration technological development and the more extreme conditions in which oil and gas companies operate (65). Previous research (9, 66, 67), hasrevealed that positive safety culture and safety communication were essential to enhanced safety performance. Therefore, the following hypotheses are established based on the preceding discussion.

**Hypothesis 2:** Safety communication has a positive impact on safety performance.

**Hypothesis 3:** Safety culture factors has a positive impact on safety performance.

### Safety Communication

Today, a growing number of businesses are prioritizing safety communications as a key value. This emphasis on safe workplace benefits not only employee morale as well as the business line. According to the “Liberty Mutual Workplace Safety Index (68),” a company may expect a $4 return on investment for every $1 invested in workplace safety. Safety communication is the most influencing tool in all aspects of business, and the workplace is no exception. Safety hazards, regulations, goals, warnings, area guidelines, rules, and progress reports should be communicated to employees via a variety of media for a truly protected workplace to be implemented (69). Organizational safety culture, leadership, and group climates are all part of the bigger picture when it comes to safety communication (70). The use of researcher-based evaluation and feedback to operational and production workers to improve their daily oral safety communication with employees resulted in considerably higher levels of safety performance in the workplace environment (71, 72). In their conceptualizations and evaluations of the safety culture in various industries, several scholars have added safety communication (73–75). The term “safety culture” refers to a set of shared organizational ideals aimed at reducing risk. This is when most people in an organization, from top leadership to temporary workers, place high importance on ensuring a safe workplace (76). Poor safety culture and communication have been linked to infamous and large-scale health and safety catastrophes in the past, demonstrating why encouraging improved safer practices is a powerful strategy (77). Employee errors, however, are not the main source of blame. Poor management actions, such as ignoring the need to develop channels of communication with employees or ignoring employee feedback, can result in large financial losses (78). Improving health and safety communication systems, such as the channels you use, how you record essential information, and employee feedback techniques has the potential to improve your company's safety culture. You will encourage greater self-responsibility in your employees as a result of this, and health and safety will become a shared obligation rather than a separated one (79). According to the finding of earlier studies (80, 81), when operational workers in the petrochemical oil and gas exploration sector in Malaysia detect a positive safety culture at the workplace, they must concentrate their efforts on performing the project rather than on safety measures, as they risk being laid off. Thus, it hypothesized that:

**Hypothesis 4:** Safety communication mediates the relationship between safety culture and safety performance.

**Figure 2:**
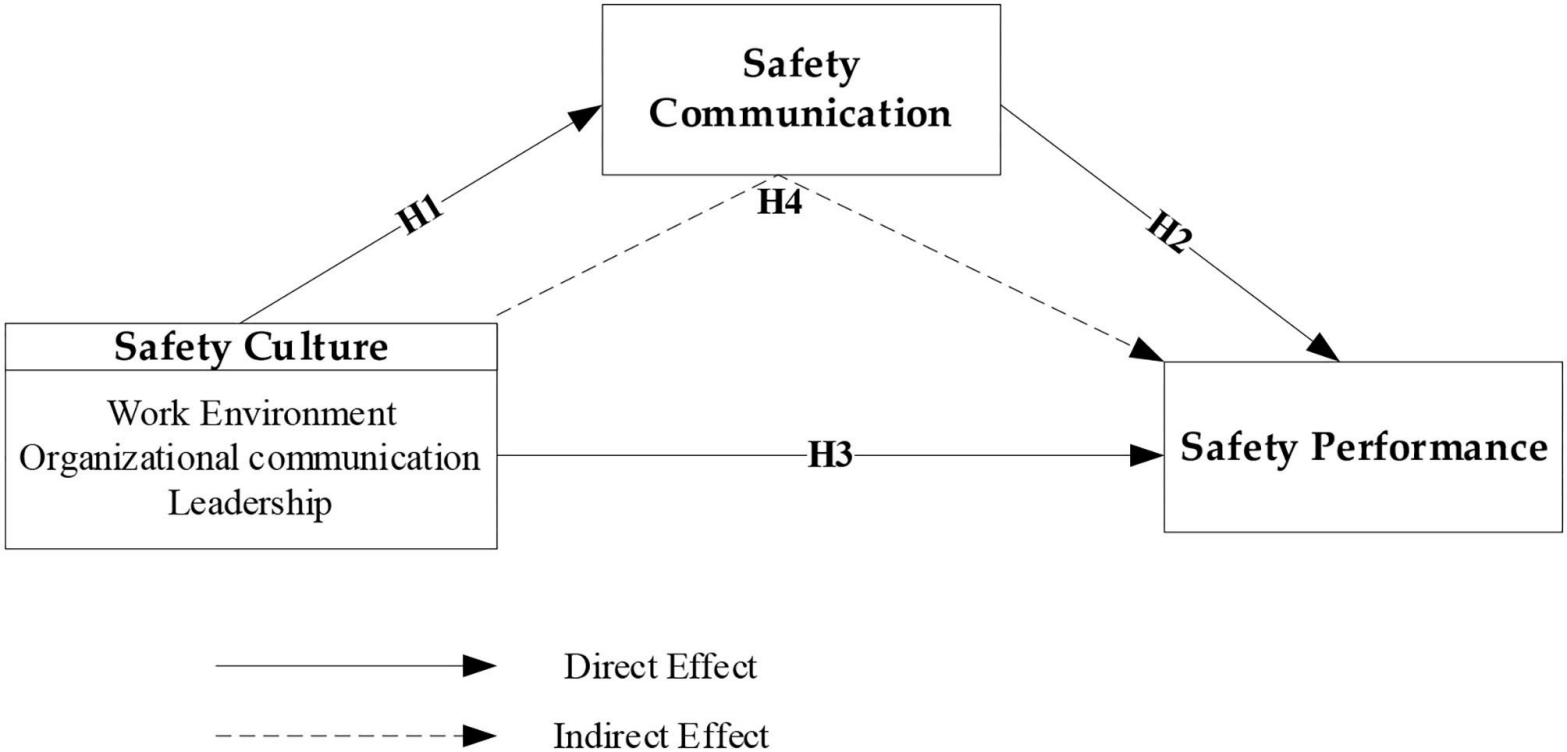
Safety performance hypothesis model.

As illustrated in Figure 2, a framework was constructed in this study to clarify that good safety performance, safety communication, and safety are all related to safety culture. This framework has highlighted three key variables that the researcher is examining (safety culture, safety communication, and safety performance).

In this study, safety culture will be an independent variable and assumed to influence safety performance (dependent variable). The connection between safety culture and safety performance will be mediated through safety communication. This research will examine the importance of safety communication in mediating the link between safety culture and safety performance in Malaysian petrochemical and oil and gas industries. By verifying the framework elements in the context of Malaysia, this study will also look at several ways for safety reducing risk, and accident prevention. The finding of this study will target to contribute to a better understanding of the factors that affect an employee's safety performance and how to increase positive safety behaviors. As a result, the model of employee safety performance will aid in the improvement of the worker's productivity and well-being, as well as ensuring his or her safety at work in general. Exchanges and protection Questionnaires based on “social exchange theory” (SET) and measuring the efficiency of exchanges have been utilized in the areas of communication, safety culture, and safety performance (83, 84).

## Methods and Materials

### Procedure

The surveys were delivered to Malaysian employees working in the petrochemical, oil, and gas industries. The principal investigator remained in the workplace throughout the questionnaire's completion to answer any queries that respondents might have. Employees in the petrochemical oil and gas sector responded with a total of 320 replies. As a result, the total sample size was set at *N* = 320. Therefore, the sample size is sufficient for 95% CI with a 5% margin of error (85).

### Measures Used

The WE, OC, LS, SCO, and SP research instruments were adapted from well-known and widely used measures by researchers in this area (attached as Appendix A). Cronbach Alpha was evaluated for all factors in a pilot test involving 60 employees. The statement was declared suitable for usage because it was over 0.70 for all the variables. The following are the instrument's specifications.

### Safety Culture

For measuring (SC), the 16-items scale was adopted by (86, 87). It has three dimensions: work environment (WE), (6 items), organization communication (OC), (5 items), and leadership (LS), (5 items). For each factor of the safety culture scale, the reliability values were determined to be as follows: work environment (0.896), organization communication (0.872), and leadership (0.922). As a result, the items are measured using a five-point Likert scale (1 = Strongly Disagree; 2 = Disagree; 3 = Neutral; 4 = Agree; 5 = Strongly Agree).

### Safety Communication

SCO was designed by Vinodkumar and Bhasi (88), to examine the level of safety communication. Comprised of five items and was used to measure safety communication at the workplace environment and enhanced the connection between safety culture and safety performance, where the reliability value was discovered (0.864). Each items were respondents answered on a five-points Likert scale (1 = Strongly Disagree; 2 = Disagree; 3 = Neutral; 4 = Agree; 5 = Strongly Agree).

### Safety Performance

For measuring (SP), the 9-item scale was adopted by Wu et al. (89). For each dimension of the safety performance scale, we tested the reliability, and the result was (0.907). Items are measured using a five-point Likert scale (1 = Strongly Disagree; 2 = Disagree; 3 = Neutral; 4 = Agree; 5 = Strongly Agree).

### Sampling and Data Collection Procedures

This research used a five-point Likert scale with 30 items as the survey questionnaire (90–92), which has been used in previous investigations (93, 94). Table 1 shows the structure of the research instruments.

**Table 1:** Instrument structure.

**Constructs**	**Dimensions**	**No. of items**	**References**
Safety culture	Work environment (WE) Organizational communication (OC) Leadership (LS)	16 6 5 5	(86, 87)
Safety performance (SP)		9	(89)
Safety communication (SCO)		5	(88)

The sample for this study was chosen using a stratified random sampling procedure from the study's population. “Stratified random sampling” is defined as a stratification or segregation procedure is followed by the selection of participants at random in every stratum (95). Total numbers of 423 questionnaires were distributed through the petrochemical oil and gas company in Malaysia. After invalid surveys were removed, 320 genuine questionnaires were obtained, with an 86.89 % response rate.

Table 2 shows the percentage of males and females as 318 (99.4%) and 2 (0.6%), respectively; the gender structure of the survey revealed a higher ratio of men than women, which is consistent with the following features of the petrochemical oil and gas companies in Malaysia.

**Table 2:** Demographic information of the respondents (*n* = 320).

**Main categories**	**Sub-categories**	**Frequencies**	**Percentages %**
Gender	Male	318	99.4%
	Female	2	0.6%
Age	20–29 Years	41	12.81%
	30–39 Years	157	49.06%
	40–49 Years	79	24.69%
	50–59 Years	35	10.94%
	60 years and above	8	2.50%
Marital status	Single	42	13.13%
	Married	265	82.81%
	Divorced	13	4.06%
Education	Graduate/Postgraduate	4	1.25%
	College	44	13.75%
	Secondary	263	82.19%
	Primary	9	2.81%

Based on marital status, respondents were married at 82.81, 13.13 % were single, and 4.06% were divorced. In terms of educational background, 82.19% of respondents had completed secondary school. Furthermore, 13.75% of the respondents had completed a college degree. In addition, 2.81% of respondents had finished their primary education, and 1.25% had a degree of graduate or postgraduate. As a result, the proportion of people with secondary education was relatively high, which corresponds to the requirements for employment in petrochemical oil & gas companies in Malaysia.

According to the frequency of the age groups in the sample, 12.81% of the total respondents are between the age of 20–29 years, 49.06% are between the age of 30–39 years, and 24.69% are between the ages of 40–49 years, and 10.94% are between the ages of 50–59 years, and 2.50% are between the ages of 60 years and above. As a result, the age composition of the sample was predominantly middle-aged and younger, which is consistent with the age requirements for employees in the petrochemical oil and gas sector.

### Structural Equation Modeling Using Partial Least Squares (PLS-SEM)

Structural equation modeling (SEM) is a multivariate method for assessing the validity of competing hypotheses and obtained samples in the context of a concept or theory (96, 97). “Partial Least Squares” & “Structural Equation Modeling” (PLS-SEM) and covariance-based structural equation modeling are the two main techniques to SEM (CB-SEM) (96, 98).

When it comes to defining the link between items and constructs for researchers, PLS-SEM is more versatile than CB-SEM (99, 100). In any sample size, PLS-SEM performswell, but it must meet the sample size's minimum criteria, which allows for the generation of variables having complex effects on particular model components. The constructs or latent variables that can be employed with both reflective and non-reflective (formative) measurement models are the focus of PLS-SEM (101).

SEM is useful in a variety of situations, including models with a large number of hidden variables and indicators. As a result, it strives for the most sparse models feasible (102, 103). SEM has been successfully applied in a variety of social science fields, including construction, industry, hotel management, competitive performance [93, 94], the environment, and organization (39).

Lastly, the four hypotheses provided in this study were evaluated using the PLS-SEM approach. Variance inflation factor (VIF) was utilized to investigate multicollinearity challenges to evaluate multi-collinearity, according to Bauer and Baumeister (104). This was performed by evaluating the measurement model's fitting and path analysis using the Smart-PLS v3.2.1 tool (105). To test common technique bias, Harman's single factor was tested using SPSS version 25.0 software.

### Normality Test

To examine the normality of the data set, a skewness and kurtosis test was carried out for all study variables. It is recommended that skewness and kurtosis range values of the variables must fall between ±1.96 (106). Both positive and negative deviations from these values are the causes of non-normality. Therefore, data (*n* = 320) was estimated for the assessment of normality assumption. It was noted that the value of Skewness and Kurtosis of all scale items was within the range of ±1.96, which is significant at 0.05. So, these results explain that data is normally distributed as shown in the table below.

**Table:** Results of normality test.

**Variables**	**Skewness**	**Kurtosis**
Safety culture	−1.116	1.841
Safety communication	−0.865	0.128
Safety performance	−0.853	1.788

## Results and Data Analysis

The suggested model was evaluated using the “Partial Least Square technique (Smart-PLS 3.2.7) software” (107). We used the recommended two-staged analytical approach to evaluate the measurement and structural models (108). “G^*^ Power version 3.1.9.2” was used to calculate the suitable sample size (109). The study's total sample size of 320 employees, comfortably exceeded the minimal sample size requirement. The number of participants in this study exceeded the recommended sample size for (PLS-SEM) analysis, which is 100 (110).

### Common Method Variance

When data is collected from a single source, it is referred to as “single-source data,” there is a possibility of common method variance (111), or if the study utilized a cross-sectional research approach (112). There is a problem with common method variance when a single component describes most of the variance in a set of data (113). Harmans' one-factor test was utilized to assess the probability of common method variance (CMV). A total of 33.54% was accounted for the single factor, showing that common factors such as technique (testing time, single sources) is not a concern for these data set. Bagozzi et al. (114) propose another method for assessing common method variance. They stated that, if the inter-correlations in a correlation matrix are much more than 0.90, there could be a problem with common method variance. Table 3 shows that the correlation matrix's values are all < 0.90. As a result, both techniques confirm that in this study, there is not a severe issue of common method variance.

**Table 3:** Discriminant validity outcomes.

	**LS**	**OC**	**SCO**	**SC**	**SP**	**WE**
LS	0.872					
OC	0.241	0.814				
SCO	0.608	0.429	0.805			
SC	0.538	0.729	0.598	0.852		
SP	0.208	0.381	0.441	0.399	0.757	
WE	0.227	0.754	0.432	0.785	0.334	0.812

*LS, Leadership; OC, Organization Communication; SCO, Safety Communication; SC, Safety Culture; SP, Safety Performance; WE, Work Environment*.

### Multicollinearity

According to Hair et al. (115), multicollinearity refers to whether independent variables (IV) in a regression model are significantly associated with each other or with the dependent variable (DV). Before hypothesis testing, multicollinearity must be established. The multicollinearity of the current investigation was confirmed using variance inflation factor (VIF) (116). There is a possibility of multicollinearity if the VIF value is larger than 3.33 (117). To investigate the issue of multicollinearity, VIF was calculated. The VIF values for safety culture and safety communication were 1.32 and 1.314, respectively, which were significantly lower than the 3.33 threshold values (117). This shows that there are no difficulties with multicellularity in the research.

### Measurement Model and Validity

There are two types of validity in the measuring model: convergent validity (CV) and discriminant validity (DV). Convergent validity describes how elements that are conceptually related are converging on the structure with which they are linked (118). Items loadings, average variance extracted (AVE), composite reliability(CR), Cronbach's alpha(CA), standardized factor loadings (SFL) are all part of the convergent validity (115). If an item's loading is equal to or > 0.7 the validity of items is accepted. But, if the items loading is <0.4 the item should be eliminated (115, 119). The items loadings (0.699–0.905) were within the permissible range. The AVE was larger than 0.5, which is consistent with the literature's indicated threshold value (115, 120–122). Where the values of SIL, CA, CR should be > 0.70 (121, 123–125). The determination coefficient *R*^2^ represents the degree of endogenous variable explained variance (99). *R*^2^ can be used to determine a structural model's explanatory capacity (126). for the development of target structures, the *R*^2^ must be acceptable, with this rage (“weak 0.25, medium 0.50, substantial 0.75”). The reliability and *R*^2^ test results are shown in Table 4.

**Table 4:** Validity, reliability and *R*^2^ values.

**Construct**.	**Items relation**	**.SFL**	**.α**	**.CR**	**.AVE**	**Value**	**R^2^ LEP”**
	**SC**		0.906	0.920	0.725	-	-
	WE1←SC	0.824					
	WE2←SC	0.865					
	WE3←SC	0.818					
	WE4 ←SC	0.832					
	WE5←SC	0.803					
	WE6←SC	0.703					
	OC1 ←SC	0.805					
	OC2 ←SC	0.810					
	OC3 ←SC	0.813					
	OC4 ←SC	0.849					
	OC5 ←SC	0.788					
	LS1 ←SC	0.847					
	LS2←SC	0.861					
	LS3←SC	0.891					
	LS4 ←SC	0.871					
	LS5 ←SC	0.876					
	**SCO**		0.873	0.920	0.637	0.358	Medium
	SCO1←SCO	0.799					
	SCO2←SCO	0.815					
	SCO3←SCO	0.794					
	SCO4←SCO	0.806					
	SCO5←SCO	0.791					
	**SP**		0.909	0.923	0.574	0.227	Substantial'
	SP1←SP	0.700					
	SP2←SP	0.776					
	SP3←SP	0.765					
	SP4←SP	0.795					
	SP5←SP	0.811					
	SP6←SP	0.792					
	SP7←SP	0.725					
	SP8←SP	0.716					
	SP9←SP	0.722					

*SFL, Standardized Factor Loadings; α, Cronbach's Values; CR, Composite Reliability; AVE, Average Variance Extracted; LEP, Level Explanatory Power*.

According to the table above, SIL values varied from (0.700 to 0.894) which are (>0.650), and CA values ranged from (0.863 to 0.908) above 0.700, CR values ranged from 0.901 to 0.924 which are (>0.650), and AVE value ranged from 0.573 to 0.726 (>0.50) (127). According to Falk and Miller (128), the *R*^2^ value ranged from 0.222 to 0.359 (≥0.10).

The degree to which constructs are different from each other is explained by discriminant validity. We used the “Fornell and Larcker”(129) criterion to determine discriminant validity. The AVE square root for all constructs must be bigger than the correlation among all other constructs (130). Table 3 reveals that all numerical values were bigger than the correlation values of all other variables, indicating that the model is discriminately valid.

### Structural Model

According to the previous research (98, 131) mentioned that, *R*^2^ values (predictive power), t-values and β- values using a “bootstrapping technique of 5,000 samples” to evaluate the structural model. The effect size f2 and Q2 values should also be reported, based on Kaufmann and Gaeckler (132). While a “*p*-value” can inform the reader whether an important impact exists, the “*p*-value” will not reveal the extent of the impact as mentioned by Sullivan and Feinn (133). Both statistically significant (*p*-value) and substantive significance (effect size) are important results to report and understand in investigations. We also used a blindfolding test to look at the model's predictive relevance (Q2), which is exclusively computed for dependence variables (DV). Q2 confirms that the observed associations are not only statistically significant but also practical, and it is only used on endogenous (dependent) construct with one or more components (134).

**Figure 3:**
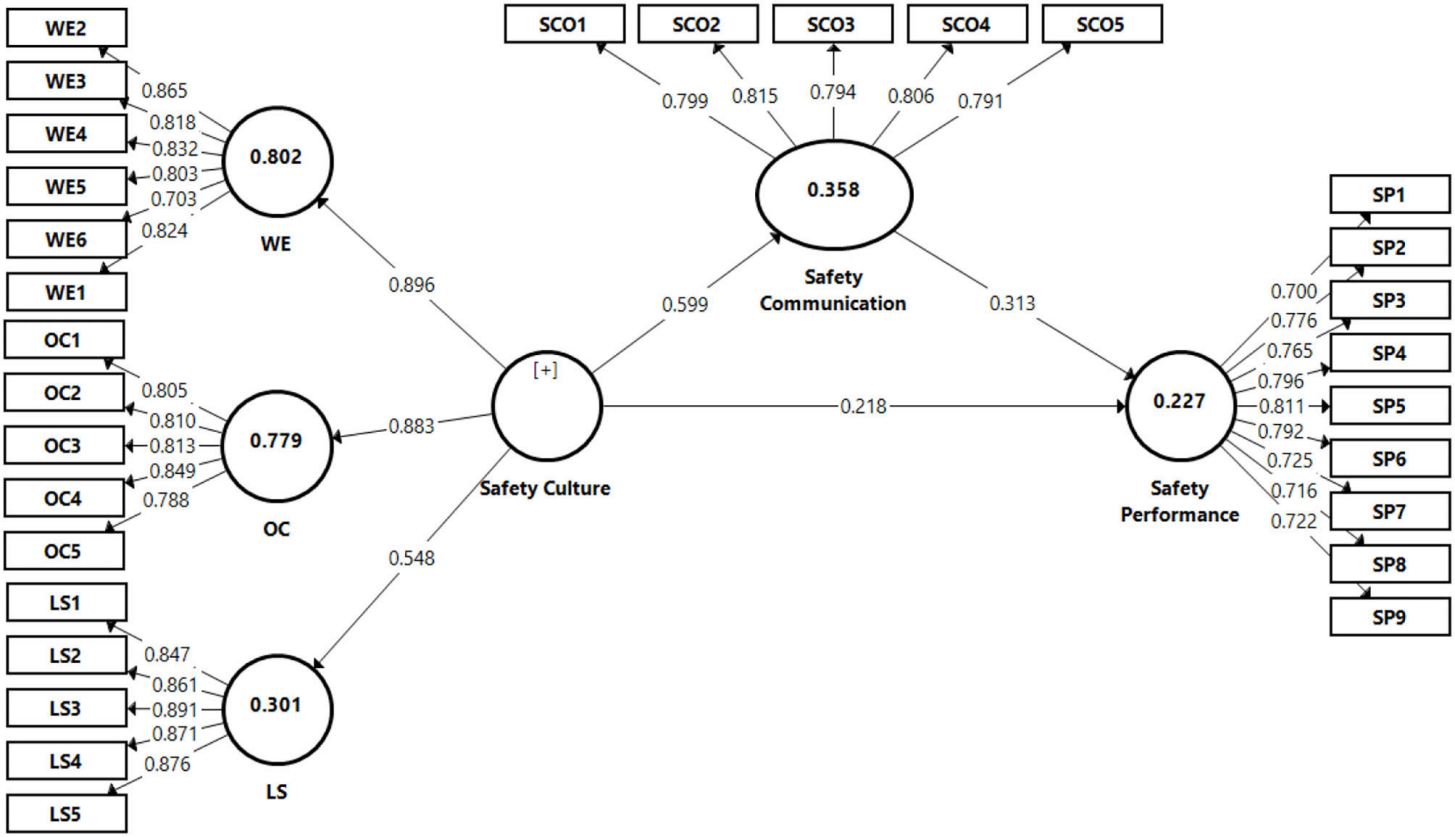
Final structural model (PLS results).

Safety communication showed mediate predictive power (*R*^2^ = 0.358). safety performance (*R*^2^ = 0.223), work environment (*R*^2^ = 0.801), organization communication (*R*^2^ = 0.532), and leadership (*R*^2^ = 0.290). For the predictive relevance (Q2) for safety communication (0.102), safety performance (0.112), work environment (0.631), organization communication (0.347), and leadership (0.028), all of the variables were higher than zero, showing that the model is predictive (115). Table 5 demonstrates the size of the effect *f*^2^ for safety culture with significant safety performance and safety communication. SC with SCO is small to mediate. The effect size for other two associations, namely safety communication and safety culture and safety performance was found small to medium (135). Three constructs were identified as independent variables (IV) under safety culture, work environment (WE), organization communication (OC), and leadership (LS), and safety performance (SP), and safety communication was the mediating variable in the final structural model. The latent variables for all of these higher-order structures were determined using a redundant indicator technique (work environment, organization communication, leadership, and safety performance) as shown in Figure 3.

**Table 5:** Findings of hypothesis testing.

**Hypothesizes**	***H1***	***H2***	***H3***
Items relations	*SC → SCO*	*SCO → SP*	*SC → SP*
Path coefficients β	0.598	0.315	0.210
STDEV	0.041	0.070	0.066
T values	14.434	4.496	3.196
*P*-values	000	000	0.002
*R* ^2^	0.358	0.223	—
f^2^	0.557	0.082	0.037
Q2	0.332	0.227	—
Significance level	***	***	***
Results	Supported	Supported	Supported

### Hypothesis Results

We anticipated a positive relationship between safety culture and safety communication. Therefore, hypothesis 1 was supported by the results (β = 0.598, *p* < 0.001). hypothesis 2 predicated that safety communication would be positively correlated with safety performance. Hypothesis 2 was supported by the results (β = 0.315, *p* < 0.001). hypothesis 3 predicted a positive relationship between safety culture and safety performance (β = 0.210, *p* < 0.001), which was supported as shown in Table 5. We used the approach given by Preacher et al. (136), to conduct the mediation analysis. According to their perspective, the indirect effect should be prioritized to achieve mediation; if it is significant, the mediation is achieved; otherwise, there is no mediation (116). Hypothesis 4 predicted that safety communication would mediate the association between safety culture and safety performance as shown in Table 6 of mediation analysis.

**Table 6:** Mediation analysis for directs and indirects effect.

**Mediation impact**	**Path coeff**	**STDEV**	***t*** **-Value**	**95% LL**	**95% UL**	**Results**
**H4**. SC-SCO-SP	0.166**	0.167	4.798	0.087	0.239	Partial mediation

If **P < 0.01; STDEV, Standard Deviation; LL, Lower Level; UL, Upper Level; H4, Hypotheses.

### Analysis of Mediation Effect

The bootstrapping method was present in this investigation to confirm the mediating impact (137). Previous research had analyzed such indirect impacts, also various researchers have used and advised this study approach (138–140). Furthermore, due to this method's aids in overcoming mediation difficulties and the lack of confidence interval of mediator and outcome variables, bootstrap findings are said to have more accurate probability estimations (141). As a result, there are two reasons why this strategy should be used. The first is that it provides a useful tool for determining the significance and confidence intervals (CI) in a variety of scenarios. When a 95% of confidence interval excludes zero, there is evidence of an indirect impact linking X to Y via a mediator, and the mediation is established. Another argument is that this method does not require a lot of assumptions. As a result, the results acquired using this method are more precise. Based on Table 6, presents the indirect effect data, which reveal that mediating hypotheses was supported.

## Discussion

The main purpose of this research was to determine the impacts of safety culture on employee's safety performance via safety communication. Based on, Social Exchange Theory (SET) (142), we examined the effect of safety communication on safety performance after developing a direct hypothesis linking safety culture to employee safety communication. It was proposed that higher levels of safety culture would allow sufficient sources to be allocated to enhancing safety performance by reducing employee accidents and fatalities. As predicted, safety communication mediated the connection between safety culture and safety performance. Safety culture, by reducing accidents, allows workers to invest their saved resources in obtaining and enhancing safety performance. Our research makes a substantial contribution to the field of theory. Firstly, it advances SC theory in the literature on occupational safety (143, 144). By demonstrating that it improves individual safety performance by reducing workplace accidents and fatalities (145). Secondly, the findings highlight the importance of enhancing the overall safety culture rather than focusing on physical safety to encourage employee's safety performance (146). It has been recommended that future research should include both safety cultures measures to enhance our understanding of their roles in workplace safety environment (147, 148), and the findings of our study recommend that future researches may require both safety culture and safety performance measures to enhance our understanding of their safety rules and regulations in the workplace place safety. Safety culture and employee safety performance were found to minimize accidents and fatalities considerably, which is consistent with previous findings (38, 61, 149, 150).

Our research adds to the safety culture literature by demonstrating that a psychosocially safe culture can prevent psychological distress and fatality even in unsafe working environments. Only a few research have looked at this association in risky conditions such as accidents and fatalities (6, 151–153). In addition, most previous research on injuries served as a measure of workplace safety, and little research has been done on how safety communication affects safety performance between workers. Our research addresses a significant gap in the literature on occupational safety and health. Based on the safety performance concept (154, 155) our research found that employees' safety performance and safety culture improves when they are improving with safety communication. Although the assessment was focused on safety leadership, it emphasized the importance of addressing the context requirements of various businesses when it came to workplace safety. Our findings revealed that in high-risk oil and gas businesses, focusing on psychological issues is just as crucial as focusing on physical factors to enhance worker safety performance. It adds to the body of knowledge in the field of occupational safety by emphasizing the significance of taking a more holistic approach. In industries where working circumstances are stressful, such as the oil and gas sector, businesses need to focus on safety cultures variables to enhance employee's safety performance. Further research into strategies to increase safety should take into account both psychosocial and physical safety. The current research examined the impact of safety culture on safety performance as a result of safety communication. The study produced numerous significant additions to the literature on safety culture in general and safety culture in particular. The study's significant theoretical achievements are listed below.

By exploring the importance of safety culture on workplace safety, the current study added to the existing body of knowledge. A theoretically recognized and recommended role (9, 156), however, little empirical work had been done to test the theoretical assumptions. Therefore, the study made a significant addition by not only experimentally proving the relevance of safety culture in safety-critical businesses like the petrochemical oil and gas industry but also establishing that it has a greater impact than safety performance. Previous studies on safety culture have usually labeled it as having a relational meaning or combining it with safety outcome behaviors (157). Because of the lack of clarity surrounding leaders' safety culture actions, many people believe that this form of leadership is useless or even harmful.

As a result, the current work contributes significantly to the literature in this area. The study's findings revealed that the effectiveness of safety culture is influenced by the context of the organization. The findings demonstrated that the impact of safety culture was much bigger than the impact of safety outcomes. The study's findings confirm that the general adoption of effective development should be approached with caution. The study contributes to the body of knowledge and lays the groundwork for future research in this area, examining the effectiveness of management communication in various organizations and cultural settings to see whether the claim of universal applicability has any validity.

The theoretical approaches of this study will serve to expand existing research and aid in the exploration of various organizational cultural situations. The research backs up the theory that learning is a social process in which employees develop behaviors dependent on how rewarding those behaviors are. The current findings show that future research into different leadership styles depending on how acceptable certain proactive behavior are in a national or corporate environment could aid in the advancement of organization theory considerably.

## Conclusion

Employee safety performances are influenced by the company's safety culture. This study verifies our assumptions that safety culture has a favorable impact on safety performance through safety communication, based on SET. The outcomes of this research add to the body of the knowledge not just in terms of the direct relationships between safety culture and safety communication, but also in terms of the indirect impact of safety culture and safety performance. This research is the first study to conceptualize and empirically examine the mediating role of safety communication between safety culture and safety performance. Furthermore, research on safety culture in Asian settings, especially in Southeast Asian countries i.e., Malaysia, is limited. This research provides strong guiding's that may be used by practitioners and academicians. These findings are explored in terms of safety interventions and future studies.

The findings have substantial consequences for professionals. Previously, positive safety culture was thought to be essential for maintaining workplace safety environments (45). However, the findings of our study suggest that to increase workplace safety organizations should focus on safety culture in addition to physical safety. Establishing a high level of safety culture at the managerial level is vital not only for psychosocial health but also for enhancing member safety performance. Constructing a work environment where workers feel their managements are committed to their psychological wellbeing, they are not overburdened with work requirements, and they are in a psychosocially safe environment reduces employee accidents, fatalities, and also stress and allows them to be more constructive inability to absorb safety information, participating in safety procedures, and putting them into action.

The safety culture literature (158, 159) explains the practical steps that must be taken to implement safety culture in organizations. Using legislation to ensure the proper application of safety culture in high-risk sectors like in oil and gas can be an efficient strategy to put safety culture rules into implementation (160). Other effective practices include safety culture in management performance appraisals and appointing people to senior roles based on their adherence to safety culture policy (161).

Finally, our findings show that high-risk companies such as oil and gas must prioritize workers' mental health such as accidents and fatalities over competing needs (such as production) to positively affect their safety performance.

### The Study Implications

The main objective of this study was to assess the impact of safety culture on the workplace safety performance indicators, i.e., leading and lagging indicators with the mediation of psychosocial hazards. We investigated the impact of the psychosocial hazard on safety performance after developing direct hypotheses concerning safety culture and psychosocial hazard. It was proposed that a high level of safety culture would enable the provision of sufficient resources to contribute toward ensuring good safety performance by reducing employees' psychosocial hazard, based on the social exchange theory (162). As expected, psychosocial hazard mediated the relationship between safety culture and safety performance. It was obvious to find psychosocial hazard's interplay between safety culture and safety performance based on conservation of resources theory (163). In doing so, by reducing psychosocial hazards for the workforce, organizations will allow their workers to invest their saved resources in learning and improving their safety habits.

Our research contributes to the field of theory in a significant way. First, it confirmed that safety culture theory promotes individual safety performance by decreasing the psychosocial hazard (164, 165). In harmony with prior literature, safety culture dimensions such as management commitment, work environment, and involvement of workers collectively and positively impact safety performance (166). Second, the findings emphasize the need to enhance the entire safety culture to promote safety performance among employees (167). Finally, safety performance was improved by reducing their indicators (leading and lagging), which can prevent any accident occurring during the workplace environment (145, 168).

In the current study, four hypotheses were tested, three hypotheses were direct predictors, and the rest utilized safety communication as a mediating relationship between safety culture and safety performance.

### Study Limitation

Because of the cross-sectional nature of our study, we must proceed with caution when interpreting our findings. A cross-sectional design was chosen for two reasons. To begin with, gathering data from the petrochemical oil and gas business was extremely challenging due to the process and time required to gain access to the local Malaysian industries. The second issue is Malaysia's lack of support for researchers prevented us from collecting data many times (169). While cross-sectional research can be useful in the early stages of a project (170), a longitudinal layout is essential to prove the causal flow and mediation. Other culture measures (particularly, safety culture) should be included in future studies to further understand their unique and comparative impacts on workplace injuries. A study on psychological wellbeing was undertaken similarly (171). The data for our analysis came from Malaysia's oil and gas industry. As a result, caution should be used when extrapolating these findings to other areas. It must be fascinating to discover if people in other industries and occupations with different levels of job requirements are affected in the same way by unsafe act unsafe conditions (UAUS). Our research did not directly assess employee resources, but it did test a theoretical framework based on the social exchange theory's premise. We suggest that a clear measure of personal resources maintained in a psychosocially and socially safe culture would contribute significantly to the safety literature and assist develop an understanding of SET. A comparative of safety performance in a safety culture “vs.” an unsafe culture could enhance safety theory while also having practical consequences for managers and organizations.

## Data Availability Statement

The original contributions presented in the study are included in the article/supplementary materials, further inquiries can be directed to the corresponding author/s.

## Author Contributions

All authors involved in the methodology design, data analysis, literature review, manuscript preparation, and have read and agreed to the published version of the manuscript.

## Conflict of Interest

The authors declare that the research was conducted in the absence of any commercial or financial relationships that could be construed as a potential conflict of interest.

## Publisher's Note

All claims expressed in this article are solely those of the authors and do not necessarily represent those of their affiliated organizations, or those of the publisher, the editors and the reviewers. Any product that may be evaluated in this article, or claim that may be made by its manufacturer, is not guaranteed or endorsed by the publisher.

## Appendix

**Table A1:** Research instrument.

**Constructs**	**Items code**	**Items**	**References**
Safety culture Work environment	WE1	“Operational targets often conflict with safety measures.”	(86 )
	WE2	“Sometimes I am not given enough time to get the job done safely.”	
	WE3	“Sometimes conditions here hinder my ability to work safely.”	
	WE4	“There are always enough people beside me to get the job done safely.”	
	WE5	“I cannot always get the equipment I need to do the job safely.”	
	WE6	“I feel safer in this place to work.”	
Organization communication	OC1	“There is good communication here about safety issues which affects me.”	(86)
	OC2	“Safety information is always brought to my attention by my line manager/supervisor.”	
	OC3	“My line manager/supervisor does not always inform me of current concerns issues.”	
	OC4	“Management operates an open-door policy on safety issues.”	
	OC5	“I do not receive praise for working safely.”	
Leadership	LS1	“My senior managers/ leaders have established a safety responsibility system.”	(87)
	LS2	“My senior managers/ leaders express an interest in acting on safety policies.”	
	LS3	“My senior managers/ leaders are concerned about safety improvement.”	
	LS4	“My senior managers/leaders establish clear safety goals.”	
	LS5	“My senior managers/ leaders coordinate with other departments to solve safety issues.”	
Safety Communication	SCO1	“My company doesn't have a hazard reporting system where employees can communicate hazard information before incidents occur.”	(88)
	SCO2	“When it comes to safety issues management has an open door approach.”	
	SCO3	“There is sufficient opportunity to discuss deal with safety issues in meetings.”	
	SCO4	“The target goals for safety performance in my organization are not clear to the workers.”	
	SCO5	“There is open communications about safety issues in this workplace.”	
Safety Performance	SP1	“He/she helps employees to recognize the importance of safety.”	(89)
	SP2	“He/she encourages employees to participate safety activities.”	
	SP3	“He/she studies new knowledge regarding safety continuously.”	
	SP4	“He/she is trying to solve the conflicts among employees.”	
	SP5	“He/she frequently communicates safety issues to employees.”	
	SP6	“He/she regularly provides employees with safety information.”	
	SP7	“You are willing to maintain the function of safety facilities.”	
	SP8	“While working it is very unlikely for you to get in contact with hazardous materials.”	
	SP9	“You clearly know the proper procedures when fire break out.”	
